# Delayed Administration of Angiotensin Receptor (AT2R) Agonist C21 Improves Survival and Preserves Sensorimotor Outcomes in Female Diabetic Rats Post-Stroke through Modulation of Microglial Activation

**DOI:** 10.3390/ijms22031356

**Published:** 2021-01-29

**Authors:** LaDonya Jackson-Cowan, Wael Eldahshan, Selin Dumanli, Guangkuo Dong, Sarah Jamil, Yasir Abdul, Waleed Althomali, Babak Baban, Susan C. Fagan, Adviye Ergul

**Affiliations:** 1 Department of Medicine, Augusta University/University of Georgia Medical Partnership, Athens, GA 30602, USA; ladjackson@augusta.edu; 2 Program in Clinical and Experimental Therapeutics, University of Georgia College of Pharmacy, Augusta, GA 30912, USA; weldahshan@augusta.edu (W.E.); walthomali@augusta.edu (W.A.); sfagan@uga.edu (S.C.F.); 3 Charlie Norwood Veterans Affairs Medical Center, Augusta, GA 30912, USA; 4 Department of Physiology, Medical College of Georgia, Augusta University, Augusta, GA 30912, USA; 5 Department of Pathology and Laboratory Medicine, Medical University of South Carolina, Charleston, SC 29425, USA; selin.dumani@mail.mcgill.ca (S.D.); jamil@musc.edu (S.J.); yasir@musc.edu (Y.A.); 6 Ralph H. Johnson Veterans Affairs Medical Center, Charleston, SC 29401, USA; 7 Department Neuroscience and Regenerative Medicine, Medical College of Georgia, Augusta University, Augusta, GA 30912, USA; gudong@augusta.edu; 8 Department of Oral Biology, Dental College of Georgia, Augusta, GA 30912, USA; bbaban@augusta.edu

**Keywords:** ischemic stroke, diabetes, neuro-inflammation, AT2 receptor, microglial polarization

## Abstract

About 70% of stroke victims present with comorbid diseases such as diabetes and hypertension. The integration of comorbidities in pre-clinical experimental design is important in understanding the mechanisms involved in the development of stroke injury and recovery. We recently showed that administration of compound C21, an angiotensin II type 2 receptor agonist, at day 3 post-stroke improved sensorimotor outcomes by lowering neuroinflammation in diabetic male animals. In the current study, we hypothesized that a delayed administration of C21 would also lower chronic inflammation post-stroke in diabetic female animals. Young female diabetic rats were subjected to 1 h of middle cerebral artery occlusion (MCAO). Three days post-stroke, rats were administered C21 or vehicle in drinking water at a dose of 0.12 mg/kg/day for 4 weeks. The impact of C21 on microglial polarization was analyzed by flow cytometry in vivo and in vitro. Compound 21 treatment improved fine motor skills after MCAO through modulation of the microglia/macrophage inflammatory properties. In addition, C21 increased M2 polarization and reduced the M1:M2 ratio in vitro. In conclusion, delayed administration of C21 downregulates post-stroke inflammation in female diabetic animals. C21 may be a useful therapeutic option to lower neuro-inflammation and improve the post-stroke recovery in diabetes.

## 1. Introduction

Stroke is a leading cause of death and disability both in the U.S. and worldwide. A vast dichotomy of stroke outcomes exists between men and women. In younger and middle-aged groups, stroke incidence rates are lower in women compared to men, whereas in the higher age groups the incidence rates in women are equal to or higher than those in men [1]. Although younger age can be protective in women, interestingly this protection is negated in the presence of diabetes [2]. Compared to diabetic males, female patients with diabetes have a higher risk of stroke [3]. Young females who survive an ischemic stroke suffer from the long-term sensorimotor and cognitive consequences of stroke. Therefore, identification of novel and safe therapeutics to aid in rehabilitation efforts for both male and female stroke victims is of critical importance.

We recently demonstrated the role that inflammation plays in stroke recovery in diabetic male animals [4,5]. There is a sustained upregulation of inflammatory cells such as microglia, macrophages and glial cells 8 weeks post-stroke in diabetes [5]. This sustained level of inflammation correlates with the poorer functional recovery experienced by these animals, as well as greater demyelination in the hippocampus and cognitive decline [5]. With microglia as the gatekeepers of inflammation within the brain, we postulated that their polarization may be a key determinant in post-stroke inflammation. Indeed, when microglia and macrophages were depleted by short hairpin RNA (shRNA)-mediated silencing of the critical colony stimulating factor 1 receptor (CSF1R), the inflammation, demyelination, functional recovery and the cognition of the animals were improved [4]. Interestingly, we have also recently demonstrated a dichotomy of post-stroke inflammatory responses in male and female animals [6]. Diabetes differentially promotes the expansion of cerebral Th17 cells in females and induces a sexually dimorphic effect on the expansion of many T-cell profiles [6]. The impact of diabetes on microglia phenotype in females before and after stroke remains to be determined, and this established the first goal of the current study.

The renin-angiotensin system (RAS) plays an essential role in the modulation of inflammation, as its receptors are present on a variety of cell types including microglia, astrocytes, neurons and oligodendrocytes [7]. AT2R stimulation with C21 is a promising therapeutic avenue in the field of ischemic stroke [5,8,9,10]. We and others have characterized the administration of C21 at reperfusion after middle cerebral artery occlusion (MCAO) with positive results [5,8,10]. We most recently demonstrated that delayed administration of C21 3 days post-stroke was able to shift the cells toward an anti-inflammatory profile. This was associated with reduced demyelination and improved cognitive recovery in male animals. Since there are multiple reports indicating sexual differences in RAS, it is important to assess AT2R stimulation in female rodents in the context of diabetes [11,12]. Accordingly, the second goal of our study was to determine the impact of delayed administration of C21 on recovery and survival after stroke in young diabetic female rats. In addition, we studied possible underlying mechanisms that could explain C21 positive effects in females. We hypothesized that administration of C21 at 3 days post-stroke improves long-term stroke outcomes in diabetic female rats through modulation of microglia/macrophage polarization toward a more anti-inflammatory profile.

## 2. Results

### 2.1. Delayed Administration of C21 Improves Survival and Sensorimotor Outcomes after Stroke

**Figure 1:**
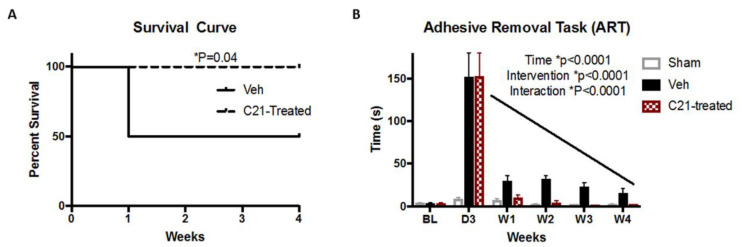
Delayed administration of C21 reduced mortality and improved functional deficits. (**A**) Vehicle-treated animals experienced a mortality of 50%, whereas C21-treated animals experienced no further mortality after day 3 (onset of C21 administration), log-rank test (*p* = 0.04). (**B**) Sensorimotor deficits were measured using the adhesive removal task (ART). No differences were observed between vehicle- and C21-treated group mortality or sensorimotor deficits at baseline. Both vehicle- and C21-treated groups displayed similar deficits 3 days after stroke and prior to C21 administration. Day 3 C21 administration enhanced recovery after a stroke. Repeated (RM) ANOVA (*p* < 0.0001 interaction), (n = 5–6/group).

It has been shown previously that C21 improves survival in males [5,13]. Furthermore, we have shown a tendency toward improvement of survival at 14 days in female Wistar rats [8]. To that end, survival was assessed in our current study. C21 significantly improved survival starting from week 1 through week 4 (Figure 1A). After assignment to treatment groups, there was no mortality in the C21 group, whereas 50% of animals assigned to the vehicle group experienced further mortality. In addition, C21 significantly lowered the time to remove the adhesive tape, indicating improvement of fine sensorimotor function. There was no difference in the latency to remove the adhesive tape before stroke, and notably both groups displayed equivalent levels of stroke severity, indicated by their similar day 3 measurements prior to vehicle and C21 treatment assortment (Figure 1B).

### 2.2. Delayed Administration of C21 Decreases Proinflammatory Microglia/Macrophages after Stroke

**Figure 2:**
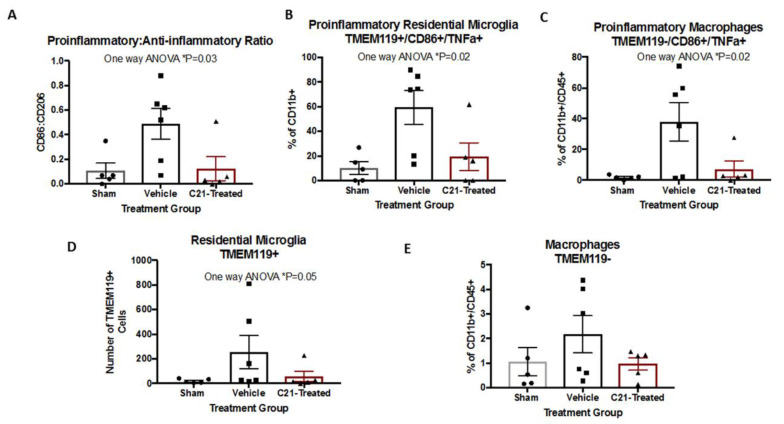
C21 may exert its beneficial effects on stroke recovery through modulation of the pro-/anti-inflammatory microglia ratio in diabetic animals. (**A**) Treatment with C21 lowered the M1:M2 ratio in female diabetic rats 4 weeks after stroke (*p* = 0.03). C21 administration lowered both the (**B**) M1 residential microglia and the (**C**) M1 macrophages (*p* = 0.02). One-way ANOVA (n = 5–6/group). (**D**) Delayed administration of C21 may act through halting the activation of residential microglia (*p* = 0.05). (**E**) It also did not impact the percentage of activated macrophages as measured by flow cytometry, as indicated in Table 1. It did however reduce the activation of residential microglia. One-way ANOVA (n = 5–6/group).

**Figure 3:**
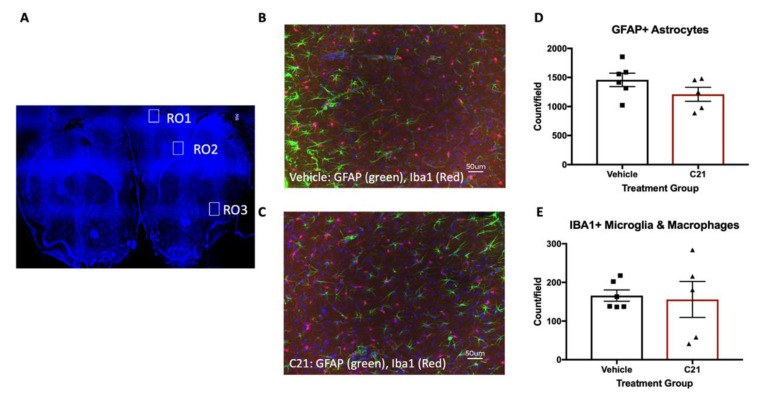
C21 did not affect the total number of microglia or astrocytes at the ischemic hemisphere. (**A**) Stitch illustration from a sham animal used to highlight the regions of interests (ROIs) in an intact brain section. Data are the average count of the 3 different ROIs in the ipsilateral RO1, 2 and 3 for GFAP+ and Iba1+ cells. Regions of interest ROI 1 and ROI 3 correspond to the neocortex, whereas ROI 2 corresponds to the corpus callosum and associated subcortical white matter. (**B**) Representative of stroked vehicle-treated animal taken from the cortex (ROI 1). (**C**) Representative of stroked C21-treated animal taken from the cortex (ROI 1) as well. C21 administration did not impact the number of GFAP+ (**D**) or Iba1+ (**E**) cells (n = 5–6/group). No significant differences were observed in individual ROs or the average ROIs across groups.

Stroke induced an increase in the ratio of the proinflammatory (M1-like) CD86+ compared to the anti-inflammatory (M2-like) CD 206+ microglia/macrophages (Figure 2A). C21 treatment decreased the pro-/anti-inflammatory microglia ratio, indicating a positive impact on stroke-induced neuroinflammation. Further analysis of microglial/macrophage subpopulation revealed a decrease in both M1 resident microglia (TMEM119^+^/CD86^+^/TNFα^+^) and infiltrating macrophages (TMEM119^-^/CD86^+^/TNF TNFα^+^) with C21 treatment (Figure 2B,C and Table 1). Notably, when evaluating the overall impact C21 administration exerted on residential microglia and macrophages, it only lowered the number of residential microglia (Figure 2D,E). This finding may be related to the chosen timepoint of 3 days post-stroke, when macrophage infiltration has already begun to occur. Next, we assessed the total number of microglia and astrocytes by immunofluorescence microscopy. C21 did not change the number of Iba1+ or GFAP+ cells in the ischemic hemisphere (Figure 3A,B). Delayed C21 administration did not impact blood glucose levels or weight gain (Figure S1).

### 2.3. C21 Treatment Induces an M2 Phenotype and Lowers the M1:M2 Ratio In Vitro in the Microglial Cell Line

**Figure 4:**
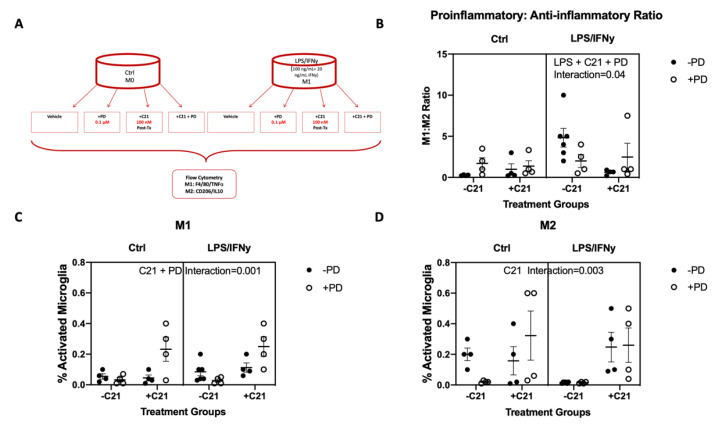
C21 lowers the pro-/anti-inflammatory microglia ratio. (**A**) C8B4 cell line was polarized toward an M1 phenotype with the incubation with both LPS and IFN*γ*. (**B**) C21 treatment improved the M1:M2 ratio in the LPS/IFN*γ* group, but only the absence of PD (interaction *p* = 0.04) (**C**) AT2R blockage with PD increased M1 microglia in both control and LPS/IFN*γ* treatment (interaction *p* = 0.001). (**D**) C21 treatment increased M2 cells in both control and LPS/IFN*γ* groups in the presence and absence of PD (interaction *p*= 0.003) (n = 4–6/group).

To determine if C21 induces an anti-inflammatory phenotype by directly acting on microglia, the C8B4 microglial cell line was treated with C21 in vitro (Figure 4A). Treatment with LPS/IFNγ resulted in an increased ratio of proinflammatory/anti-inflammatory markers (Figure 4B). Delayed C21 treatment 6 h post-LPS/IFNγ exposure at a dose of 100 mM decreased the M1:M2 ratio only in the absence of AT2R blockage with PD (interaction *p* = 0.004). C21 treatment did not alter the M1 cells, but increased the M1:M2 ratio by increasing M2 microglia (interaction *p* = 0.003; Figure 4C,D). PD blockage in C21 treatment halted the impact of C21 on the M1:M2 ratio by increasing the M1 microglia without altering the M2 ratio (interaction *p* = 0.001; Figure 4C,D).

### 2.4. Stroke Did Not Significantly Alter IL-17 Levels from Microglia

**Figure 5:**
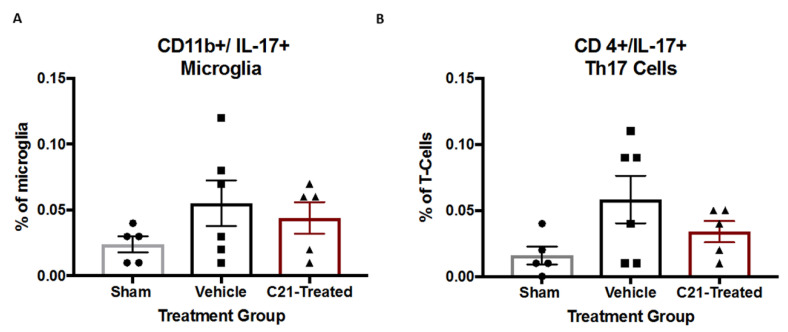
C21 did not alter IL-17 levels in the ischemic hemisphere. (**A**) C21 administration did not alter IL-17-producing microglia (Iba1+/Il-17+). (**B**) C21 did not alter CD4+/Th17 cells. One-way ANOVA (n = 5–6/group).

We previously showed that diabetes and stroke increased the percentage of IL-17-producing T cells (Th17) within the T cell population in the brains of diabetic rats with and without stroke [6]. Therefore, we set out to determine the impact of stroke and C21 treatment of diabetic rats on the levels of IL-17 producing cells such as IL-17-producing microglia and Th17 cells. Stroke did not significantly change the levels of IL-17-producing microglia (Figure 5A) or T cells at 4 weeks post-stroke (Figure 5B). Post-hoc analyses indicated a trend toward an increase in Th17 cells in vehicle-treated animals compared to the shams, which was not present in the C21-treated animals.

### 2.5. At a Dose of 0.03 mg/Kg/Day Delayed Administration of C21 Does Not Significantly Alter Cognition in Female Rats

**Figure 6:**
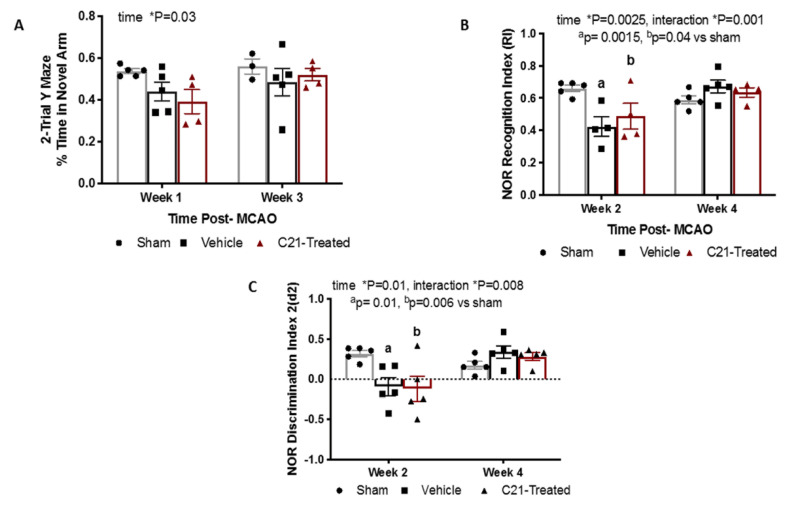
C21 did not impact cognition in female rats. (**A**) C21 did not affect spatial memory as assessed by 2 trial Y-maze. (**B**) C21 did not affect recognition memory as assessed by novel object recognition (NOR) testing. (**C**) No differences were observed among groups. One-way ANOVA.

Our lab and others have reported that HFD/STZ-induced diabetes results in cognitive impairment in female rats [5,6,14,15,16,17]. We also reported that delayed administration of C21 in male rats preserves cognition post-stroke [5,9]. To study the impact of stroke and C21 on cognition in diabetic females, both spatial memory and recognition memory were assessed using 2-trial Y-maze and novel object recognition (NOR) tests, respectively (Figure 6A,B). A decline in memory was observed in weeks 1 and 2 post-stroke as measured via Y-maze and NOR, respectively, yet no notable differences between vehicle- and C21-treated groups were observed. No memory impairment was observed at weeks 3 and 4 in either group.

## 3. Discussion

The objectives of the current study were to determine whether delayed administration of C21 prevents sustained inflammation and improves chronic recovery after stroke in female diabetic animals, and to elucidate possible underlying mechanisms. In the current study, C21 was administered at 3 days post-stroke to study its effect on secondary neurodegenerative processes well outside the neuroprotective window after transient MCAO. The infarct development after transient MCAO in rat brains reach a maximum volume at 3 days post-stroke [18]. This is consistent with clinical studies in which a peak infarction is reached at 3 days, followed by a continuous decline in infarct volume [19]. We have previously demonstrated the neuroprotective effect of C21 given at reperfusion in control male and female rats [8,10]. In the current study, delayed administration of C21 on day 3 post-stroke in diabetic female rats improved survival and sensorimotor outcomes at 4 weeks post-stroke, similar to what was observed in male animals 8 weeks post-stroke [5]. We set out to determine fine motor impairments after stroke using the adhesive removal task (ART), which detects subtle differences in motor outcomes. C21 decreased the latency to remove the adhesive tape, indicating that fine motor skills are preserved with C21 treatment. Due to the high mortality after stroke in diabetes experienced in our previous studies and the increase in mortality experienced in vehicle-treated diabetic female animals, we limited this study to 4 weeks post-stroke. Although this is an earlier timepoint, it is encouraging to see a similar effect on survival and sensorimotor outcomes.

The improvement in stroke outcomes is associated with a C21-induced increase in anti-inflammatory and decrease in pro-inflammatory microglia/macrophages, which is also similar to what we observed in male diabetic animals. This was incredibly reassuring to observe, as it suggests that although sex differences in the RAS system exist, RAS therapeutics may exert beneficial effects in both sexes. In our past study, C21 treatment prevented further decline in recognition and spatial memory after stroke in male diabetic rats, beginning at 1 week post-stroke and continuing out to 8 weeks post-stroke [5]. In the current study in diabetic females, we did not see an effect of C21 on cognition. This is intriguing, since female brains have been shown to have more angiotensinogen-positive neurons than males and we thus expected a greater impact on cognitive outcomes [7]. There are several possibilities that may have resulted in this outcome. First, this could be attributed to the impact of hormonal fluctuation, which has been reported to affect some cognitive assessments such as NOR [20]. In an effort to synchronize the female estrous cycle, we introduced male urine-soiled bedding in the cages of female rats (Whitten effect) as previously described [21,22]. We also observed that this cohort of animals behaved differently during the tests. They were agitated and tried to jump out of the NOR field or Y-maze set-ups. The use of male bedding may have caused this and affected their performance on cognition tests. It was demonstrated previously in female rats that different phases of the estrous cycle affect behavioral indices of anxiety [23,24,25]. Previous studies have also demonstrated that male bedding can induce estrus in female animals, a portion of the cycle characterized by high estrogen levels. This may have also contributed to the quick recovery observed in the animals, with cognitive deficits no longer present at 3 weeks of age. It is also possible that the effect of C21 on the cognition of intact females is not robust at the administered dose or appears at a later time point. In an animal model of multiple sclerosis in females, C21 was used with success at a dose of 0.3 mg/kg, which is 10 times higher than the dose used in the current study [26]. It is also worth noting that the post-stroke cognitive deficits observed in the vehicle-treated animals were very mild, pointing again to possible hormonal influences. However, D3 ART scores suggest significant deficits and stroke severity not being a factor in this lack of progressive cognitive decline. Lastly, in our previous study using male diabetic animals, we observed that the initial (2 weeks post-stroke) decline in cognitive function after stroke improved by week 4, although after this point there was a progressive decline in diabetic but not control animals. In the current study we also saw a trend for decline at week 2 as measured by NOR which recovered by week 4. As mentioned above, due to greater mortality in female diabetics, we followed these animals only for 4 weeks after stroke, but longer follow-up may reveal the impact of C21 on cognition.

We previously reported memory impairment in diabetic male and female rats using both embolic and suture models of MCAO [17]. The impairment was evident as early as 7 days post-stroke and was associated with neurodegeneration and an increase in hyperreactive microglia in CA1 and DG regions of the hippocampus [17]. Although C21 has been shown in multiple studies to improve spatial memory, in this current study it relieves the pro-inflammatory burden without impacting cognition [27,28]. Although we previously highlighted the role of microglia in the development of post-stroke cognitive impairment in male animals, this difference highlights the possibility that additional modulators may be in play in female animals. Emerging evidence suggests that there is functional and regional microglial heterogeneity [29,30,31]. As such, different microglial subtypes may respond differently to any intervention in females, and this deserves further exploration.

There is evidence that T lymphocyte infiltration increases infarct volume and worsens stroke outcomes [32,33]. Interleukin-17 (IL-17) is secreted from differentiated T helper-17 (Th17), cells which is a subset of CD4+ T cells [34]. IL-17 blocking antibody decreased infarct size and improved neurological outcomes poststroke. A similar effect was observed when IL-17 was knocked out [35]. Lastly, we previously demonstrated that diabetes deferentially upregulates Th17 cells in diabetic females but not males [6]. These data suggest that IL-17 inhibition is critical in the acute period post-stroke and may be deferentially regulated in a males and females. In the current study, we only observed a nonsignificant increase in CD4^+^/IL-17^+^ at 4 weeks post-stroke, suggesting that IL-17 does not play a major role beyond the acute phase of stroke. Therefore, our delayed administration of C21 after the acute period may have halted its ability to mediate this measure. This additionally suggests that the differential regulation of IL-17 cells in the acute phase may be a key difference in the development of post-stroke cognitive impairment in diabetic male and female animals.

Since previous studies have illustrated the key role that microglia play in post-stroke recovery, to study the underlying mechanism of C21 actions, we focused on the direct effect on microglia. C21 decreased pro-inflammatory microglia and infiltrating macrophages at 4 weeks post-stroke without an effect on the total number of Iba1+ cells. This is similar to what was observed in males and suggests that C21 exerts its effects through the modulation of phenotype rather than the downregulation of microglia [5]. The anti-inflammatory impact of C21 on microglia was also observed in vitro in the presence of LPS/IFNγ, indicating that C21 exerts direct actions on microglia and in corroboration with previous reports indicating the presence of RAS receptors on microglia [7]. We also observed similar effects of C21 on microglia in earlier studies in hypertensive and diabetic males using two different methods of microglial analysis [5,9]. In these studies, microglial phenotype was assessed using either histologic morphometric methods or by flow cytometry. It is important to note that while many of the beneficial effects of C21 in vivo are modulated by AT2R, we have also shown that the direct modulation of microglia with C21 in cell culture is independent of AT2R stimulation [5]. In this study we discovered that although the M1:M2 ratio remains unchanged, blockage of AT2R resulted in increased M1 polarization with C21 treatment.

One possible mechanism by which C21 improves the M1:M2 ratio is through BDNF secretion. We previously demonstrated that stimulation of the AT2 receptor increases the expression of BDNF in brain tissue after experimental ischemic stroke and in brain microvascular endothelial cells, resulting in improved outcomes and a proangiogenic state, respectively [36,37]. We also have shown that BDNF expression is a major contributor to the positive effects of RAS modulation in experimental stroke [38]. It is likely that many cell types of the neurovascular unit, including pericytes, astrocytes and neurons, express BDNF however, and their relative contributions to functional recovery is difficult to discern [39]. Microglia have also been shown to secrete BDNF, especially after stimulation with LPS [40,41]. Further studies are warranted to elucidate the dependence of AT2R stimulation on BDNF secretion, as well as the role of acute C21 administration on Th17 cells and cognitive impairment in female animals.

In summary, we report that AT2 receptor stimulation with compound 21 in diabetic female rats improves survival and fine sensorimotor skills post-stroke. The mechanism of C21′s actions could involve polarizing microglia/macrophages toward an anti-inflammatory phenotype. We recognize the limitations in the low numbers of animals and the relatively short duration of post-stroke monitoring for cognitive deficits. Nevertheless, our findings have high translational value since female patients with comorbidities such as diabetes tend to suffer the most from stroke. Future pre-clinical studies should include comorbid models and longer monitoring times to successfully assess post-stroke cognitive impairment and stroke recovery. Our results will help in the design of future pre-clinical studies in this regard.

## 4. Materials and Methods

### 4.1. Animal Model

Female Wistar rats (Envigo RMS, Inc., Indianapolis, IN, USA) were housed in the animal care facility at Augusta University, which is approved by the American Association for Accreditation of Laboratory Animal Care. All experiments were conducted in accordance with the National Institute of Health (NIH) guidelines for the care and use of animals in research. Furthermore, all protocols were approved by the Augusta University institutional animal care and use committee (protocol number 2013-0573, approval date 08/03/2016).

### 4.2. Middle Cerebral Artery Occlusion (MCAO) Surgery

Female diabetic animals were subjected to transient focal cerebral ischemia (60 min MCAO) or sham surgery at 14 weeks of age using a 4-0 silicon-coated nylon suture (Doccol 403756 or 403534), depending on the rat size. The animals that weighed 350–425 g received the 403756 suture, whereas animals weighing 300–350 g received the 403534. This was optimized prior to the start of the study to result in similar infarct sizes across weight ranges. The animals were anesthetized using 2–5% isoflurane, a ventral mid-line neck incision was made, the right common carotid artery (CCA) was exposed and lightly tied, and the external carotid artery (ECA) was ligated and cut. The suture was marked at 1.8 and 2 mm then advanced from a nick at the ECA into the internal carotid artery (ICA) until being positioned in between the 1.8 and 2-mm marks, indicating the branching of the MCA. The suture was tied in place for the duration of the occlusion and the animals were allowed to recover from anesthesia. At the end of the 60-min occlusion time, the animals were re-anesthetized, the suture was removed for reperfusion and the small nick at the ECA was permanently ligated. In sham surgeries, the CCA was isolated and the ECA was cut and ligated without insertion of the suture. Animals were first randomized to sham (n = 5) and MCAO (n = 14) surgery. Three animals in the MCAO group died prior to randomization to vehicle (n = 6) or C21 (n = 5) treatment groups, blinded as groups A or B on day 3 post-MCAO. After randomization, group A, which turned out to be the vehicle group after unblinding at the end of the study, experienced greater mortality within the first days of assignment into the group. Due to this, extra animals were added (n = 6) to group A to account for the difference. The mortality was 50% in this group. Group B, which turned out to be the C21 group, experienced 0% mortality. Final animal numbers include sham (n = 5), vehicle (n = 6) and C21 treatment (n = 5). In the post-operative period, blood glucose (BG) was monitored daily for 7 days and then weekly.

### 4.3. Treatment and Behavioral Assessments

#### Dose and Timing Justification

We chose day 3 to start administering C21 because it is well out of the neuroprotective window of 6 h and acute infarct evolution is likely to be complete. The oral dose of 0.12 mg/kg was calculated based on an oral bioavailability of 0.25, and was thus equivalent to an intravenous (IV) dose of 0.03 mg/kg [10].

### 4.4. Blinding and Randomization

The treatment and vehicle groups were prepared by an individual not involved in the surgery or assessments and labeled as group A and group B. Each animal was numbered before baseline behavioral assessments were taken. After MCAO surgery, the animals were assigned to group A and B using a random number generator. All behavioral and histological assessments were coded and conducted by a blinded investigator. Drinking water groups were also blinded.

### 4.5. Cycle Synchronization

In an effort to synchronize cycles within the cohort of animals, cages were changed to include male bedding for a total of 4 weeks before surgeries and for the duration of the remaining experiment. In an effort to minimize the impact of cycle phasing, the surgeries were conducted over a period of 3 days.

### 4.6. Assessment of Sensorimotor Function

Sensorimotor function was evaluated by means of ART as previously described [16]. The animals were trained for 4 days and then baseline measurements were recorded prior to stroke. Subsequent ART measurements were recorded at weeks 1, 2 and 4 post-stroke. Contact and removal latency of the adhesive paper dot was recorded and the average was taken from 3 trials with a maximum removal latency of 180 s per trial.

### 4.7. Assessment of Cognitive Function

Cognition was assessed by means of 2-trial Y-maze and novel object recognition (NOR) tests. The animals were trained 4 days prior to baseline testing. Testing was conducted at baseline prior to stroke, followed by weeks 1 and 3 for the Y-maze, and weeks 2 and 4 for NOR. The 2-trial Y-maze was used to examine spatial memory. In the first trial, animals were allowed to freely explore 2 open arms for 10 min. The animal was returned to its home cage for a 15-min delay. In the second trial, animals were allowed to explore all 3 arms of the Y-maze apparatus freely for 3 min. Total time spent in each arm was recorded. Results were expressed as % time spent in the novel arm (time in novel arm divided by total time in all arms × 100). NOR was utilized to examine working memory. On testing days, each rat participated in 3 phases: habituation, familiarization and novel. In the habituation phase animals were allowed to explore for 5 min and then returned to its home cage for a 15-min delay. In the familiarization phase rats were allowed to explore 2 identical objects placed equidistant from the walls with 20 cm between the objects for 5 min. After a 45 min delay in their home cage, each rat was allowed to explore a novel object paired with the familiar object for 5 min. Rats were started in the center of the testing apparatus for each session. Objects and the testing area were cleaned with vital oxide odor eliminator between each phase. The time spent exploring each object was recorded to calculate recognition index (RI; RI = (TN)/(TN+TF)) and discrimination index 2 (d2 = (TN- TF)/ (TN+TF)).

### 4.8. Euthanasia, Specimen Collection and Molecular Techniques

Animals were euthanized 4 weeks post-stroke or sham surgery using isoflurane overdose and cardiac puncture. Coronal sections were prepared using a brain matrix as described previously [5]. Sections were taken for flow cytometry and immunocytochemistry. Regions of interest ROI 1 and ROI 3 corresponded to the neocortex, whereas ROI 2 corresponded to the corpus callosum and associated subcortical white matter.

### 4.9. Flow Cytometry

The prefrontal cortex and hippocampus were isolated through separation of the B and D slice. The tissue was then minced into 1 mm^3^ pieces and was dissociated using Worthington’s Papain Dissociation kit (catalog number LK003153) with the following modifications: (1) tissue was left in dissociation medium for 15–25 min and (2) oxygen was continuously perfused over (not bubbled within) the solution for the duration of the incubation period [42]. Microglia were isolated as described below.

### 4.10. Myelin Debris Removal and Microglial Isolation

A debris removal step was performed using modified protocols from Miltenyi Biotec’s Myelin Removal Kit (catalog number (Miltenyi Biotec, Gladbach, Germany) and CD11b^+^ Microbeads (Miltenyi Biotec, Gladbach, Germany). Following dissociation, up to 10^7^ cells were suspended in 200 μL 0.5% BSA in PBS buffer and incubated with 20 μL anti-myelin microbeads for 15 min at 4 °C. The cells were then placed in the mini-MACS magnetic separator column and the clean supernatant was eluted out. The cells were then incubated with 20 μL of CD11b^+^ beads to isolate the microglia/macrophage population and isolated using the mini-MACS separator once again. CD11b^+^ cells were then further processed with surface and intracellular microglia makers.

### 4.11. Cellular Staining

Cells were incubated with surface markers against pre-conjugated antibodies CD45-APC (ebioscience, San Diego, CA, USA) and CD86-FITC (BD bioscience, San Jose, CA, USA) for 20 min. Cells were then permeabilized for intracellular staining with a fixation/permeabilization solution kit (ebioscience, San Diego, CA, USA). Cells were then separated into two groups and incubated with markers CD206 (Abcam, Cambridge, MA, USA) or TNFα (BD bioscience, San Jose, CA, USA) and TMEM119 (Novus, Centennial, CO, USA). Secondary antibodies PE (eBioscience, San Diego, CA, USA) and PerCP (BD bioscience, San Jose, CA, USA) were used in both groups. Cells were then washed and analyzed using the Cytoflex (Beckman Coulter, Indianapolis, IN, USA).

### 4.12. Imaging and Analysis

To minimize false-positive events, the number of positive events, detected with the negative staining control for each individual channel, was subtracted from the number of positive cells stained with corresponding antibodies. Cells expressing a specific marker were reported as a percentage of the number of gated events. The gating strategy is shown in Table 1. Microglia were first identified as CD11b^+^/CD45^+^ low. Proinflammatory microglia were further identified as CD86^+^; anti-inflammatory cells were identified as CD206^+^. Residential microglia versus infiltrating macrophages were also identified as CD11b^+^/CD45^+^ without separation of low versus high. Residential microglia were then further identified as TMEM119^+^, whereas infiltrating macrophages were identified as TMEM119^-^. M1 macrophages were then identified as CD11b^+^/CD45^+^/TMEM119^-^/CD86^+^/TNFα^+^ cells.

### 4.13. Immunohistochemistry (IHC)

Brains were extracted and post-fixed in 4% PFA overnight. Free-floating 30-μm sections were incubated overnight with anti-iba1 (ionized calcium-binding adaptor molecule 1, 1:500, Wako, Japan) and anti-GFAP (glial fibrillary acidic protein, 1:400, Sigma-Aldrich, Burlington, MA, USA) for D slice sections containing the hippocampus. Cells were then incubated with Texas red and Alexa Flour 488-conjugated secondary antibodies (Cell Signaling Technology, Danvers, MA, USA) used at 1:200 for 2 h at room temperature. Nuclei were counterstained using Dapi (406-diamidino-2-phenylindole, Roche, Basel, Switzerland) and sections were mounted. Imaging was performed using the Keyence Microscope (Itasca, IL, USA) and Z stacked through the 30-μm thickness at a 1-μm pitch to obtain a complete count of the tissue area for iba1 and GFAP quantifications.

### 4.14. Cell Culture

The direct effect of C21 on microglia polarization was determined in mouse cells (C8B4) using flow cytometry-based analysis of polarization markers. Cells were treated with LPS (Lipopolysaccharide, 100 ng/mL) and IFNγ (interferon γ, 20 ng/mL) to induce an inflammatory phenotype (F4/80^+^/TNFα^+^). Cells were post-treated with C21 (100 nM) with or without the AT2R blocker PD 123319 (0.1 μM) 6 h post LPS/IFNγ exposure to evaluate whether C21 impacts inflammatory (F4/80^+^/TNFα^+^) or anti-inflammatory (F4/80^+^/CD206^+^/IL-10^+^) polarization.

### 4.15. Statistical Analyses

Prism 7 GraphPad (www.graphpad.com) was used to analyze all data. Data with three groups were analyzed by one-way ANOVA (Figure 2, Figure 3, Figure 5 and Figure 6). Data with 8 groups were analyzed by three-way ANOVA (Figure 4). Survival data were analyzed by a log-rank test. Repeated measure ANOVA was used to analyze ART data (Figure 1). Data are presented as mean ± SEM. A *p* value of <0.05 was considered significant.

## Figures and Tables

**Table 1:** Flow cytometry markers utilized to identify particular cell populations.

Cell Types	CD11b	CD45	TMEM119	CD86	TNFα	CD206	IL-17	CD4
M1(CD86^+^/TNFα^+^)	+	+low	N/A	+	N/A	N/A		
M2(CD206^+^/IL10^+^)	+	+low	N/A	-	N/A	+		
Residential Microglia (TMEM119^+^)	+	+	+	N/A	N/A	N/A		
Infiltrating Macrophages	+	+	-	N/A	N/A	N/A		
M1 macrophages	+	+	-	+	+	N/A		
Inactivated microglia	+	+low	N/A	-	N/A	-		
IL17^+^ Microglia	+	+low	N/A	N/A	N/A	N/A	+	N/A
Th17	N/A	N/A	N/A	N/A	N/A	N/A	+	+

## Data Availability

The data presented in this study are available on request from the corresponding author. The data are not publicly available due to federal guidelines.

## Supplementary Materials

Supplementary materials can be found at https://www.mdpi.com/1422-0067/22/3/1356/s1. Figure S1: Delayed C21 administration did not impact blood glucose levels or weight gain. Weight loss after stroke was also similar in vehicle- and C21-treated groups, suggesting that stroke severity was comparable between the groups.

## Abbreviations

AT2R	Angiotensin II type 2 receptor
BDNF	Brain-derived neurotrophic factor
TH17	T helper 17
MCAO	Middle cerebral artery occlusion
ART	Adhesive removal test
