# Supplementary material for: Delayed Administration of Angiotensin Receptor (AT2R) Agonist C21 Improves Survival and Preserves Sensorimotor Outcomes in Female Diabetic Rats Post-Stroke through Modulation of Microglial Activation
Source: Int J Mol Sci. 2021 Jan 29;22(3):1356. doi: 10.3390/ijms22031356 (PMC7866408; doi:10.3390/ijms22031356)
Supplement: Supplementary file 1 [file ijms-22-01356-s001.pdf]

International Journal of
Molecular Sciences

MDPI

Supplementary Materials

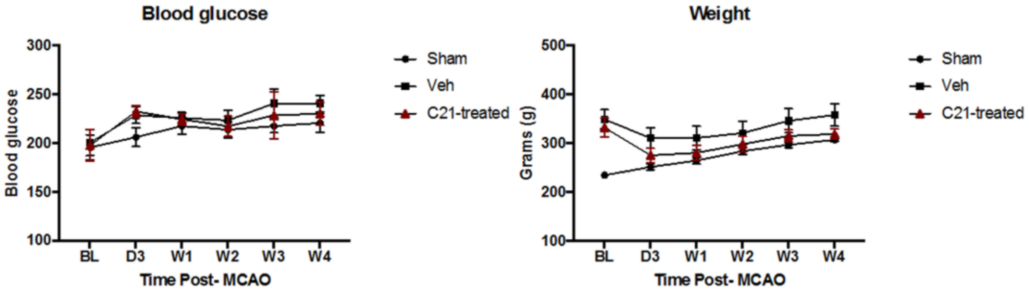

Figure S1. Delayed C21 administration did not impact blood glucose levels or weight gain. Weight loss after stroke was also similar in vehicle and C-21 treated groups suggesting that stroke severity was comparable between the groups.

© 2021 by the authors. Submitted for possible open access publication under the terms and conditions of the Creative Commons Attribution (CC BY) license (http://creativecommons.org/licenses/by/4.0/).
